# Highly sensitive interleukin 6 detection by employing commercially ready liposomes in an LFA format

**DOI:** 10.1007/s00216-021-03750-5

**Published:** 2021-11-13

**Authors:** Simone Rink, Barbara Kaiser, Mark-Steven Steiner, Axel Duerkop, Antje J. Baeumner

**Affiliations:** 1 Institute of Analytical Chemistry, Chemo- and Biosensors, University of Regensburg, Universitätsstraße 31, 93053 Regensburg, Germany; 2 Microcoat Biotechnologie GmbH, 82347 Bernried am Starnberger See, Germany

**Keywords:** Lateral flow assay, Point-of-care diagnostics, Bioanalysis, Fluorescence liposomes, Colloidal gold, Interleukin 6

## Abstract

Recent years have confirmed the ubiquitous applicability of lateral flow assays (LFA) in point-of-care testing (POCT). To make this technology available for low abundance analytes, strategies towards lower limits of detections (LOD), while maintaining the LFA’s ease of use, are still being sought. Here, we demonstrate how liposomes can significantly improve the LOD of traditional gold nanoparticle (AuNP)–based assays while fully supporting a ready-to-use system for commercial application. We fine-tuned liposomes towards photometric and fluorescence performance on the synthesis level and applied them in an established interleukin 6 (IL-6) immunoassay normally using commercial AuNP labels. IL-6’s low abundance (< 10 pg mL^−1^) and increasing relevance as prognostic marker for infections make it an ideal model analyte. It was found that liposomes with a high encapsulant load (150 mmol L^−1^ sulforhodamine B (SRB)) easily outperform AuNPs in photometric LFAs. Specifically, liposomes with 350 nm in diameter yield a lower LOD even in complex matrices such as human serum below the clinically relevant range (7 pg mL^−1^) beating AuNP by over an order of magnitude (81 pg mL^−1^). When dehydrated on the strip, liposomes maintained their signal performance for over a year even when stored at ambient temperature and indicate extraordinary stability of up to 8 years when stored as liquid. Whereas no LOD improvement was obtained by exploiting the liposomes’ fluorescence, an extraordinary gain in signal intensity was achieved upon lysis which is a promising feature for high-resolution and low-cost detection devices. Minimizing the procedural steps by inherently fluorescent liposomes, however, is not feasible. Finally, liposomes are ready for commercial applications as they are easy to mass-produce and can simply be substituted for the ubiquitously used AuNPs in the POCT market.

**Graphical abstract:**

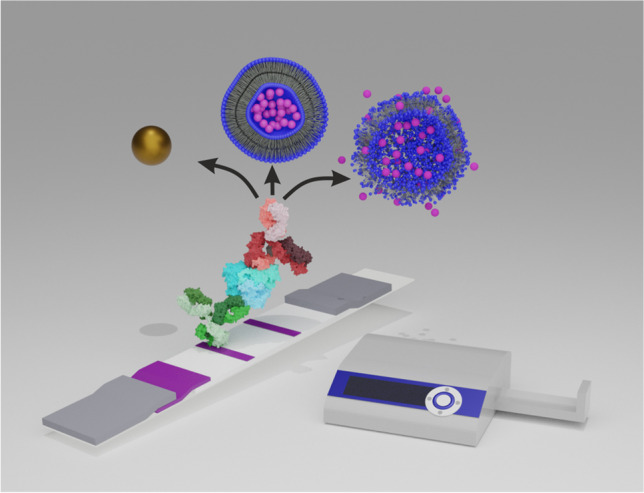

**Supplementary Information:**

The online version contains supplementary material available at 10.1007/s00216-021-03750-5.

## Introduction

Not only the global pandemic in 2020 emphasized the need and relevance of simple, fast, sensitive, and one-site point-of-care (POC) solution in the medical diagnostic field, but also a market size of USD 29 billion in 2020 (which is estimated to rise to USD 67 billion in 2026, www.reportsanddata.com) [1] depicts its global interest. In this fast-growing market, lateral flow assays (LFA) belong to the major gameplayers, as they are typically very fast and offer low costs and straightforward operation even by non-experts [2]. Aside from the numerous benefits of LFAs, such as amenability to inexpensive mass production, autonomy of additional external equipment, and optical readout, in its simplest form with the human eye, standard LFA platforms still have to face limitations with regard to sensitivity due to their mostly semi-quantitative nature and often provide users only with yes/no answers. A lateral flow assay is typically conducted in either a competitive or sandwich assay format [3]. Here, the test strip consists of a test line typically utilizing a biomolecule directed against the analyte as a capture probe and a control line which is directed against the reporter particle. The sample is added to the sample pad and resolubilizes the reporter particles such as gold nanoparticles (AuNP) or colored latex beads from the subsequent conjugate pad along its capillary force–driven flow throughout the test strip [4]. Depending on the applied format, the analyte-reporter complex binds to the test line yielding an increasing signal with increasing analyte concentration in a sandwich assay format. The test strip consists of various porous materials with each having its unique feature, i.e., assisting in sample transport, containment, and homogenous release of reagents, ensuring homogenous fluid flow, and capturing relevant biomolecules through capture probes via precise test line manufacturing. This general design of the LFA allows vast room for sensitivity enhancement at several stages of the LFA development. Bishop and colleagues [4] critically assessed the adjusting screws which have recently been studied to push the development and potential of lateral flow assays towards sensitivity levels similar to those of laboratory-based test systems. Especially, the applied reagents and envisioned reactions and their realization on an LFA were thoroughly discussed. Key factors for enhancing the sensitivity of an LFA by several orders of magnitude are the development of high-affinity reagents, tweak of transport dynamics for ideal reaction kinetics as well as label and detection optimization of conventional reporter particles, or even integration of signal amplification strategies [4]. Ultimately, investigating a combination of these individual strategies is of special interest. In this study, we focused on the reporter particles and signal amplification strategies by exchanging conventional AuNP with fluorescent dye–loaded liposomes. Liposomes are mainly known as delivery vehicles in medicine and pharmacology but paved their way into analytical and bioanalytical applications as detection particles due to their comparably high surface area, a large internal volume, and flexible surface modification with various biorecognition elements [5, 6]. IL-6 is an important biomarker for immune response and inflammatory processes in the human body. It belongs to the class of pro-inflammatory cytokines currently under evaluation inter alia as a potential biomarker to identify COVID-19 positive patients who are at risk of respiratory failure and death due to severe inflammatory response [7, 8]. Its growing diagnostic relevance as a prognostic marker for infections, especially due to COVID-19, and its presence in low pg mL^−1^ concentrations in serum [9] (in healthy subjects < 10 pg mL^−1^) make it an ideal candidate for this study. We herein study sulforhodamine B (SRB)–loaded liposomes with different sizes in a sandwich-based LFA for the detection of interleukin 6 (IL-6) in direct comparison to conventional AuNP. These liposomes were synthesized entrapping SRB, a highly water-soluble fluorescent dye allowing encapsulation of e.g. up to 1.2 million molecules of a 150 mmol L^−1^ dye solution in a single 300 nm liposome [6] enabling visual and fluorescence readout possibilities when applied to an LFA [10]. The accompanied signal amplification and their double readout feature rise interest in these liposomes for ultrasensitive detection in lateral flow assays. Although these particles have been applied to lateral flow assays previously [10–14], these publications only exploit the colorimetric readout possibility of liposomes focusing on the academic point of view, as the fluorescence of SRB is quenched in intact liposomes. The flexible nature of liposomes with regard to encapsulated dye, size, and surface modification gives additional advantages over standard AuNP such as e.g. multiplexing. We herein demonstrate the evolution of these liposomes to commercially ready detection particles and the gain in sensitivity by applying liposomes with optimized size in direct comparison to a commercial standard AuNP approach and designed for colorimetric and fluorescence readout. Sensitivity improvement by one order of magnitude was already obtained for the colorimetric readout with 350nm-sized liposomes whereas fluorescence measurement can significantly enhance the resolution due to an extraordinary gain in signal intensity. In addition, we show that our protein-modified liposomes remain highly stable for long-term storage in solution and also when dehydrated to the conjugate pad for a ready-to-use LFA. This renders the here demonstrated IL-6 liposome–based LFAs as a model system for any AuNP-based LFA that requires significantly lower LODs to become relevant as POCTs.

## Experimental section

### Chemicals and consumables

All chemicals were commercial analytical reagent grade and were used without purification. Phospholipids, 1,2-dipalmitoyl-sn-glycero-3-phosphocholine (DPPC), 1,2-dipalmitoyl-sn-glycero-3-phospho-(1′-rac-glycerol) (sodium salt) (DPPG), and 1,2-dipalmitoyl-sn-glycero-3-phosphoethanolamine-*N*-(glutaryl) (sodium salt) (*N*-glutaryl-DPPE), 1,2-dipalmitoyl-sn-glycero-3-phosphoethanolamine-*N*-(biotinyl) (sodium salt) (biotinyl-DPPE) were purchased from Avanti Polar Lipids (Alabaster, AL, USA); 4-(2-hydroxyethyl)piperazine-1-ethanesulfonic acid (HEPES), purity > 99.5%, was purchased from VWR chemicals (Germany). Sulforhodamine B (SRB) (230162, 75%) and *N-*hydroxysuccinimide (NHS) were purchased from Sigma-Aldrich/Merck (Germany). Fetal bovine serum (FBS) (10270–106) was purchased from ThermoFisher Scientific (Germany), and human serum (HS) was provided by Microcoat Biotechnology GmbH (Bernried, Germany). 2-(*N*-morpholino)ethanesulfonic acid (MES) and bovine serum albumin (BSA) (T844.2) were obtained from Carl Roth (Karlsruhe, Germany). Custom-made lateral flow test strips as well as anti-digoxigenin and anti-IL-6 conjugates were kindly provided by Microcoat Biotechnology GmbH (Bernried, Germany) as well as recombinant human IL-6 (200–06, Peprotech, Germany), sheep anti-digoxigenin Fab (11214667001 (Roche) Sigma-Aldrich, Germany), LFA running buffer (Art. No. 850003) and LFA 5 X serum buffer (ESS-2913). For all experiments, ultrapure water was used. A more detailed list of standard chemicals and consumable is given in the SI.

### Synthesis of sulforhodamine B liposomes

Liposomes, containing sulforhodamine B, with 6 mol% carboxy functionalization, were prepared according to an established protocol from Edwards et al. [15] with slight adjustments. Shortly, encapsulant was prepared by dissolving sulforhodamine B (150 mmol L^−1^) in 4.5 mL 0.02 mmol L^−1^ HEPES buffer, pH 7.5. DPPC (29.58 mg, 40.3 µmol), DPPG (15.64 mg, 21.0 µmol), cholesterol (19.99 mg, 51.7 µmol) and *N*-glutaryl-DPPE (6.2 mg, 7.0 µmol) were dissolved in 3 mL chloroform and 0.5 mL methanol and thoroughly sonicated in an ultrasonic bath (VWR ultrasonic cleaner, model USC 300 THD) at 60 °C. Subsequently, 2 mL of preheated (60 °C) encapsulant was added to the lipid solution and emulsified for 4 min at 60 °C, using an ultrasonic bath. After emulsification, the residual solvent was evaporated at 60 °C under reduced pressure. The remaining 2 mL of encapsulant was added after gradual evaporation to 780 mbar and thoroughly vortexed before evaporation was continued to 400 mbar. The remaining solution was extruded at 60 °C successively through 1.0 µm, 0.4 µm, and 0.2 µm membrane using a mini extruder (Avanti Polar Lipids, Inc.) to obtain unilamellar liposomes. Purification was first performed by size-exclusion chromatography with Sephadex® G-50 as stationary phase (column size: 2 cm × 8 cm) and HSS buffer (10 mmol L^−1^ HEPES, 200 mmol L^−1^ sodium chloride, 200 mmol L^−1^ sucrose, 0.01 wt% sodium azide), pH 7.5, osmolality 0.643 osmol kg^−1^ as mobile phase. Additionally, the liposomes were dialyzed against HSS buffer until the dialysis buffer remains colorless, pH 7.5, osmolality 0.643 osmol kg^−1^ before the determination of the hydrodynamic diameter via DLS, phospholipid concentration via ICP-OES, and zeta potential was done.

### Dynamic light scattering (DLS) and ζ-potential measurements

Dynamic light scattering (DLS) and ζ-potential measurements were done on a Malvern Zetasizer Nano-ZS (Malvern Panalytical, Germany). For all measurements, the temperature was set to 25 °C. Size determination was done in semi-micropolymethyl methacrylate (PMMA) cuvettes (Brand, Germany), and ζ-potential was done in disposable folded capillary zeta cells (Malvern Panalytical, Germany). The liposomes were diluted at 1:100 and measured in HSS buffer with the following settings: refractive index (RI) of the material of 1.34, material absorbance of zero, and RI of 1.342 of the dispersant viscosity of 1.1185 mPa s applied for DLS. For ζ-potential, a refractive index of 1.342, a viscosity of 1.1185 mPa s, and a dielectric constant of 78.5 were used. An equilibration time of 60 s was applied before each measurement.

### Phospholipid concentration

Phospholipid concentration was determined through inductively coupled plasma optical emission spectrometer (ICP-OES) measurements with a SPECTROBLUE TI/EOP (SPECTRO Analytical Instruments GmbH, Kleve, Germany). Phosphorous was detected at 177.495 nm and the device calibrated between 0 and 100 µmol L^−1^ phosphorous in 0.5 mol L^−1^ HNO_3_. Before each measurement, the device was recalibrated with 0.5 mol L^−1^ HNO_3_ and 50 µmol L^−1^ phosphorous. A 1:150 dilution of the liposomes (3 mL) in 0.5 mol L^−1^ HNO_3_ was measured. ICP-OES measurements yielded a phospholipid concentration of 6.7 ± 0.04 mmol L^−1^.

### Protein coupling to liposomes

The *N*-glutaryl modified liposomes were mixed with EDC (10 mg mL^−1^ in 0.05 M MES buffer, pH 5.5) and NHS (10 mg mL^−1^ in 0.05 M MES buffer, pH 5.5) and incubated for 1 h at room temperature (RT) while shaking. The respective equivalent of protein (1 mg mL^−1^ in PBS) was added and incubated for 1.5 h at RT while shaking. A ratio of 1:17:42:0.017 (n(COOH):n(EDC):n(NHS):n(protein)) was applied for antibody coupling. For streptavidin, a ratio of 1:100:180:0.23 was used. Lysine-HCl (1 mol L^−1^ in ultrapure water) was added to yield a final concentration of 10 mmol L^−1^ and again incubated for at least 10 min at RT while shaking to quench the reaction. The conjugated liposomes were purified via size exclusion chromatography using Sepharose CL-4B as stationary phase and HSS buffer (10 mmol L^−1^ HEPES, 200 mmol L^−1^ sodium chloride, 200 mmol L^−1^ sucrose, 0.01 wt% sodium azide), pH 7.5 as mobile phase, and a flow rate of approximately 0.5 mL min^−1^. The conjugates were subsequently characterized by optical density, hydrodynamic diameter, and zeta potential measurements. Optical density was measured at 565 nm of a 1:100 dilution in demineralized water. DLS and zeta potential measurements were done on a Malvern Zetasizer Nano-ZS (Malvern Panalytical, Germany). For all measurements, the temperature was set to 25 °C. The liposomes were diluted 1:500 in demineralized water and measured with the following settings: refractive index (RI) of the material of 1.45, material absorbance of 0.001, and RI of 1.330 of the dispersant viscosity of 0.8872 mPa s applied for DLS. For ζ-potential, a refractive index of 1.330, a viscosity of 0.8872 mPa s, and a dielectric constant of 78.5 were used. An equilibration time of 60 s was applied before each measurement.

### Lateral flow assay procedure

The LFA with all reagents in solution was performed according to the following procedure if not stated differently. Thirty-six microliters of the respective dilution of recombinant IL-6 in serum and 9 µL serum buffer were mixed with 5 µL detection solution consisting of detection particles (colloidal gold and 350 nm liposomes (30 mOD per test), 190 nm liposomes (40 mOD per test)) and anti-IL-6 IgG-biotin (50 ng per test) and incubated 5 min at RT in a 2 mL reaction vessel prior to application on the test strip. After 15 min, the test strip was evaluated with a ESEQuant LFR strip reader. The test strip consists of a transparent backed CN150 (colloidal gold and 190 nm liposomes) or CN95 (350 nm liposomes) nitrocellulose membrane (Sartorius, Göttingen, Germany) with a streptavidin (SA) test line (27 mm) and an anti-mouse IgG control line (36 mm). The LFA with the reagent solution applied on the test strip was done by adding the reagent solution (5 µL detection particles (colloidal gold and 350 nm liposomes (30 mOD per test), 190 nm liposomes (40 mOD per test)) and anti-IL-6 IgG-biotin (50 ng per test) to the overlap of sample pad and conjugate pad and placed directly into 45 µL IL-6 dilution including 9 µL serum buffer. The LFA was allowed to run for 15 min and was evaluated photometrically directly after the test run or allowed to dry before lysis with 2 µL absolute ethanol for fluorescence evaluation. Photometric detection was done directly after a test run *λ*_max_ = 520 nm; fluorescence was measured before and after lysis of the dried strip with 2 µL absolute ethanol with *λ*_ex_ = 470 nm, *λ*_em_ = 600 nm if not stated otherwise.

### Apparatus

Fluorescence measurements were performed with a Synergy Neo 2 microplate reader from BioTek (Bad Friedrichshall, Germany) for fluorescence measurements of liposomes or with the ESEQuant LFR strip reader from Qiagen (Hilden, Germany) for photometric and fluorescence measurements of the test strips.

## Results and discussion

### Development of photometric and fluorescent liposomes

In view of a commercial application of liposomes in lateral flow assays, we studied modifications of previously reported SRB liposomes [10] on the synthesis level towards long-term stability in solution and dehydrated on a test strip. We designed them for excellent photometric and fluorescent performance. In theory, the larger the liposome, the more encapsulant is present and hence can contribute to signal recording. Also, the small size distribution of a liposome population enhances their colloidal stability during long-term storage. However, the synthesis of large unilamellar liposomes with small size distribution is difficult [16] and not that applicable to the POCT as it typically involves additional procedural steps and can become quite time-consuming which rapidly increase the cost. We therefore chose the reverse-phase evaporation method that is known for high encapsulation yields followed by size extrusion to quickly generate differently sized liposomes in a very simple, mass-producible manner. Furthermore, by varying the encapsulant concentration, we tailored the liposomes towards photometric (high SRB concentrations) or inherent fluorescent (low SRB concentrations that do not self-quench) detection strategies. A mandatory design feature is the creation of single-step LFA procedures to maintain the dramatic advantage LFAs have over other POCT systems.

#### Inherently fluorescent liposomes

We developed inherently fluorescent liposomes by reducing the amount of encapsulant dye. It is known that 150 mmol L^−1^ SRB liposomes exhibit fluorescence self-quenching in their intact state [10]. Hence, high-performance fluorescence measurement with these liposomes is only possible through the release of SRB by lysis of the liposomes. Consequently, the fluorescence signal of such intact liposomes is typically below 1% of the fluorescence of its lysed pendant. While significant signal enhancement could be achieved, in a lateral flow assay, this would be accompanied by an additional lysis step. Instead, liposomes which are already fluorescent in their intact state avoid any additional procedural step and are thus desirable for enhanced fluorescent LFA designs. Liposomes with 10, 50, and 150 mmol L^−1^ SRB maintained self-quenching. For these liposomes, the fluorescence intensity of the intact liposomes remained below 1% (Tables 1 and 2, *I*_intact_ (%)). However, the overall fluorescence performance (*I*_lysed_) of the different batches when being lysed continuously decreases with decreasing encapsulant concentrations as expected (Table 1).

**Table 1:** Characteristics of small sulforhodamine B liposomes (extruded through 0.2 µm membrane)

Encapsulant concentration	Hydrodynamic diameter^a^ (nm)	ζ-potential (mV)	Polydispersity index	*I*_lysed_^b^ × 10^3^ (a. u.)	*I*_intact_^c^ (%)
0.1 mmol L^−1^	141 ± 40	− 37 ± 3	0.06 ± 0.01	0.059 ± 0.002	103 ± 4
1 mmol L^−1^	136 ± 46	− 39 ± 4	0.09 ± 0.01	1.25 ± 0.03	10.5 ± 0.2
10 mmol L^−1^	116 ± 51	− 32 ± 2	0.06 ± 0.01	8.1 ± 0.2	0.76 ± 0.02
50 mmol L^−1^	197 ± 68	− 30 ± 2	0.10 ± 0.01	15.9 ± 0.3	0.204 ± 0.004
150 mmol L^−1^	204 ± 61	− 31 ± 2	0.07 ± 0.01	39.5 ± 0.9	0.204 ± 0.005

^a^Size by intensity of a 1:100 dilution; ^b^*I*_intact_ was obtained by diluting liposomes to 100 µmol L^−1^ total lipid in HSS buffer (100 µL) and *I*_lysed_ by diluting the liposomes in 30 mmol L^−1^ *n*-octyl-*β-D*-glycopyranoside in HSS buffer; ^c^*I*_intact_ = *I*_intact_/*I*_lysed_ × 100, data are presented as mean ± SD with *n* = 3

**Table 2:** Overview of fluorescence intensities of large and small sulforhodamine B liposomes

Large liposomes^a^	*I*_lysed_^b^ × 10^3^ (a. u.)	*I*_intact_^c^ (%)	Small liposomes^d^	*I*_lysed_^b^ × 10^3^ (a. u.)	*I*_intact_^c^ (%)
10 mmol L^−1^	12.5 ± 0.5	0.66 ± 0.03	10 mmol L^−1^	8.1 ± 0.2	0.76 ± 0.02
50 mmol L^−1^	49.3 ± 0.9	0.259 ± 0.005	50 mmol L^−1^	15.9 ± 0.3	0.204 ± 0.004
150 mmol L^−1^	66 ± 1	0.152 ± 0.002	150 mmol L^−1^	39.5 ± 0.9	0.204 ± 0.005

^a^350 nm liposomes; ^b^*I*_intact_ was obtained by diluting liposomes to 100 µmol L^−1^ total lipid in HSS buffer (100 µL) and *I*_lysed_ by diluting the liposomes in 30 mmol L^−1^ *n*-octyl-*β-D*-glycopyranoside in HSS buffer; ^c^*I*_intact_ = *I*_intact_/*I*_lysed_ × 100; ^d^190 nm liposomes, data are presented as mean ± SD with *n* = 3

**Fig. 1:**
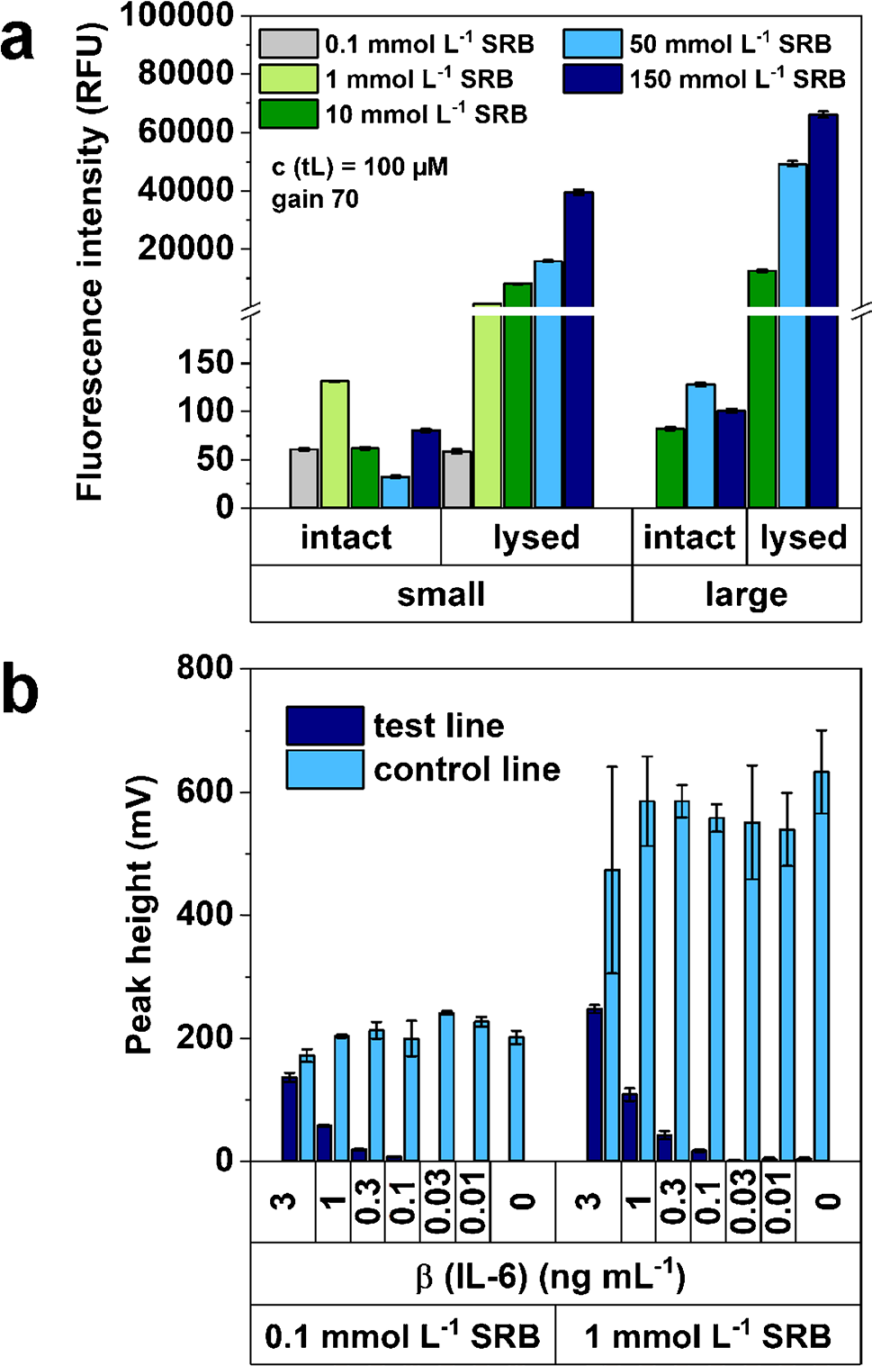
Fluorescence performance of large and small liposomes with varying encapsulation concentrations. **a** Fluorescence performance of intact and lysed large and small liposomes in solution of 100 µL liposome dilution (*c* (total lipid) = 100 µmol L^−1^) in HSS buffer (intact) or 30 mmol L^−1^ *n*-octyl-*β*-*D*-glucopyranoside in HSS buffer (lysed) and **b** fluorescence performance of intact small 0.1 and 1 mmol L^−1^ SRB liposomes in an IL-6 LFA, data are presented as mean ± SD (error bar) with *n* = 3; fluorescence signal was recorded with *λ*_ex_ = 550 nm, *λ*_em_ = 600 nm

Only the liposomes with 0.1 and 1 mmol L^−1^ SRB showed increased fluorescence > 1% in their intact state, yet their overall fluorescence performance (*I*_lysed_) in solution was very poor with as little as three orders of magnitude lower signals when lysed (59 RFU for the 0.1 mmol L^−1^ SRB liposomes in comparison to the 39,500 RFU for 150 mmol L^−1^ SRB liposomes). Thus, while the low-concentrated encapsulated dye prevented its self-quenching, it consequently reduced the obtained lysed fluorescence signal, too (Fig. 1 a). When using both inherent fluorescence liposomes in an interleukin 6 LFA test (Fig. 1 b), it was found that the overall lower fluorescence signal outweighs the positive effect obtained through sensitive fluorescence detection. Specifically, consistent with the observations in solution, both liposomes performed poorly in their fluorescent LFA. Furthermore, in comparison to the photometric detection of the high encapsulant equivalents (150 mmol L^−1^), both 0.1 and 1 mmol L^−1^ SRB liposomes are by a factor of 10 less sensitive in their fluorescent IL-6 assay.

#### Optimization of photometric liposome detection

To fully harness the liposome signaling capability for the photometric approach, they were maximally loaded with SRB by increasing their size and thus the inner volume to increase their overall sensitivity. Furthermore, we were seeking to generate liposomes with small size variation to increase their colloidal stability during long-term storage. Liposomes can be synthesized using the reverse-phase evaporation method and extruded to a desired size range through various sized membranes and extrusion steps (see Table S1 and Fig. S1) which is further supported by Szoka and colleagues [17]. Based on this information, we developed large liposomes in the range of 350 nm (PdI: 0.18) (Table S2) by extrusion through only the 1 µm membrane and small liposomes in the range of 190 nm (PdI: 0.07) (Table 1) by extrusion through 0.4 and 0.2 µm membranes with varying SRB encapsulation concentrations. These liposomes showed in initial characterizations that the larger the liposome and the higher the SRB concentration is, the more SRB is encapsulated within the liposome, as evidenced by the fluorescent measurement of lysed liposomes (*I*_lysed_) (Table 2). As these small and large 150 mmol L^−1^ SRB liposomes yielded the strongest fluorescence signal and are easy to manufacture, these two types of liposomes were chosen to evaluate their tolerance towards antibody coupling and dehydration and determine their overall performance in a regular LFA. Even larger liposomes could be investigated in the future; however, based on prior experiences (data not shown), it is assumed that more steric hindrance and less colloidal stability could hamper large liposomes.

### Photometric and fluorescence lateral flow immunoassay

Previously optimized conditions for coupling streptavidin to liposomes were not directly transferrable to IgG coupling [15]. Thus, we used the relatively inexpensive anti-digoxigenin IgG (< Dig >) to identify the ideal coupling ratio of 1:17:42:0.17 (n(COOH):n(EDC):n(NHS):n(antibody)) and obtained already in this experiment a 4-times steeper slope and an order of magnitude lower detection limit of 1 ng mL^−1^ *vs.*10 ng mL^−1^ with our liposome approach in contrast to commercial AuNPs (Online Resource 1 – ESM 2.3, Fig. S2).

These coupling conditions were subsequently used for the covalent attachment of anti-interleukin 6 (< IL-6 >) to the liposomes using a coupling ratio of 1:17:42:0.017 (n(COOH):n(EDC):n(NHS): n(antibody)) for anti-interleukin 6 (< IL-6 >). Two different antibodies were tested, where one antibody showed an over 10-times higher sensitivity with over 20-times stronger signals especially for low concentrations (data not shown) which was consequently used in the following experiments.

**Fig. 2:**
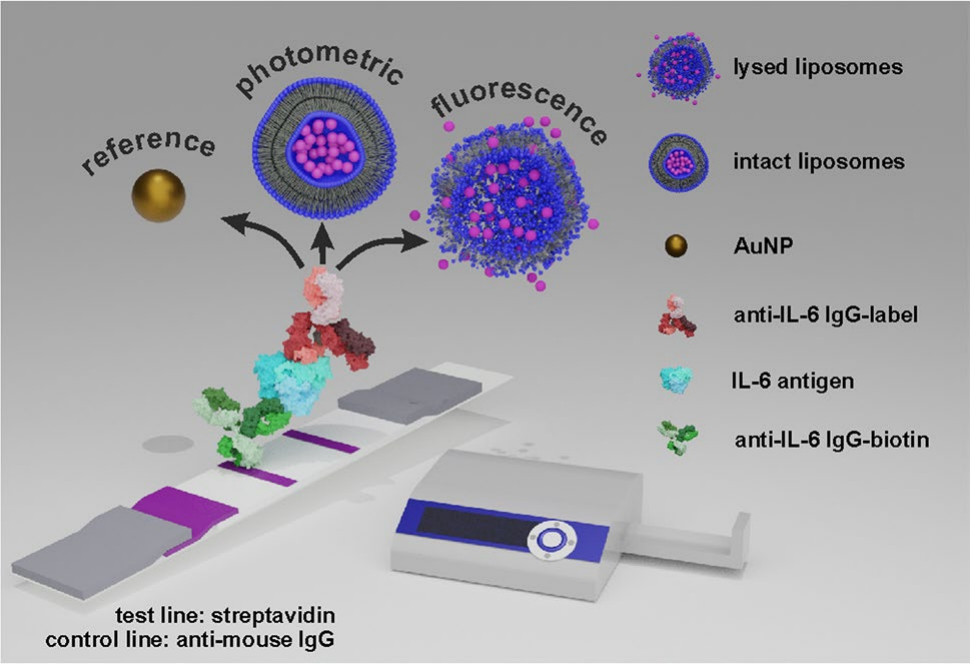
Illustration of applied analysis principle of developed interleukin 6 lateral flow assay

**Fig. 3:**
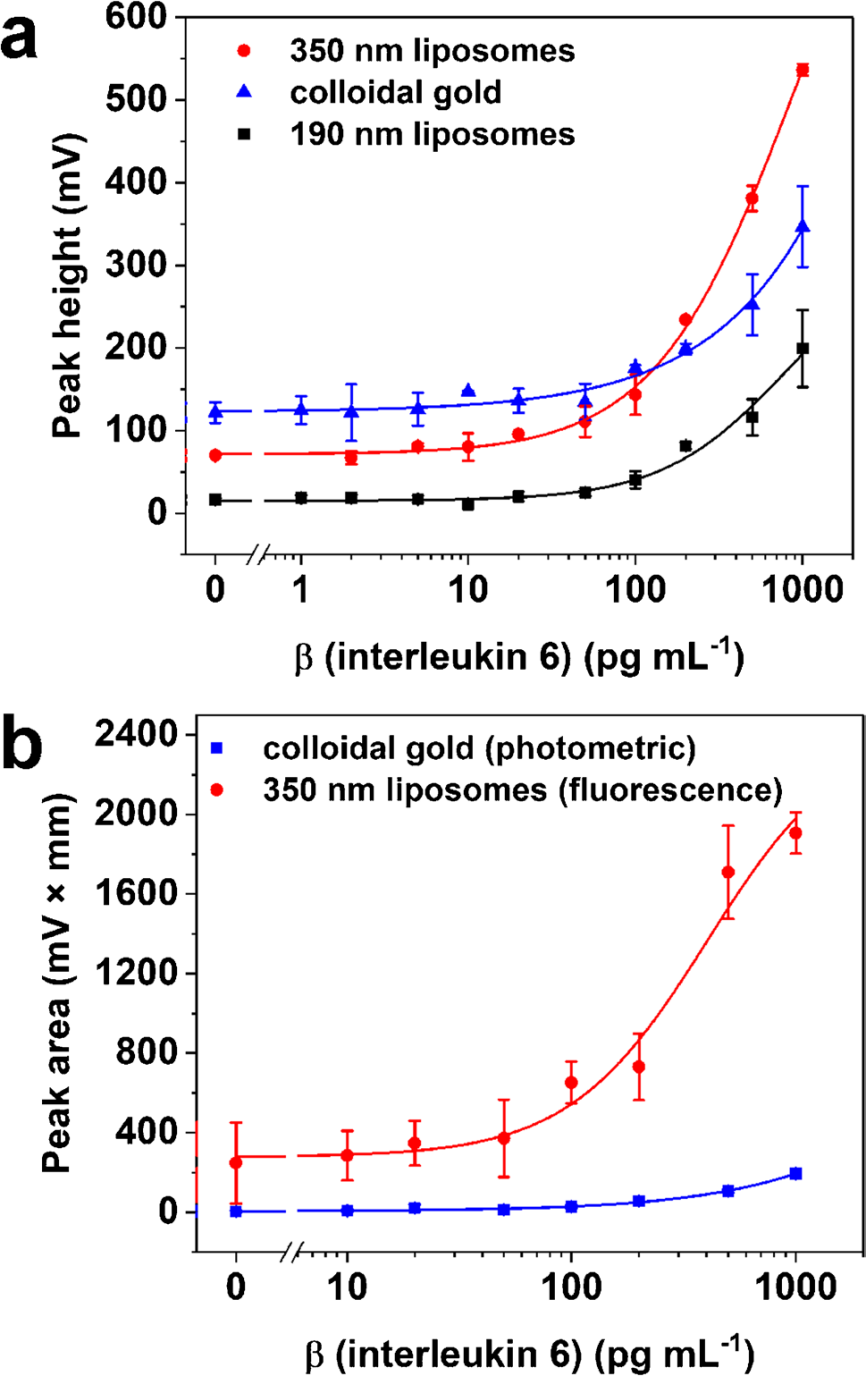
Titration of IL-6 with large and small liposome conjugates benchmarked to colloidal gold. **a** Photometric detection in human serum and **b** fluorescence detection of liposomes after lysis in human serum benchmarked to colloidal gold (photometric detection). In **a**, preincubation of liposomes (5 min) with IL-6 and anti-IL-6-biotin IgG in running solution; in **b**, liposomes on conjugate pad without preincubation. Photometric measurement was done at *λ*_max_ = 520 nm; fluorescence signal was recorded with *λ*_ex_ = 470 nm, *λ*_em_ = 600 nm; data are presented as mean ± SD (error bar) with *n* = 3; four-parameter logistic fitting with Origin2020 was done within **a** *R*^2^ = 0.9940 (red), *R*^2^ = 0.9687 (blue), and *R*^2^ = 0.9557 (black) and in **b** *R*^2^ = 0.9493 (red) and *R*^2^ = 0.9583 (blue), *y*_LOD_ = A1 + 3 SD_blank_

In the end, small and large liposomes were coupled accordingly to clone 2 (< IL-6 >) and tested in the IL-6 LFA format towards the standard colloidal gold approach as illustrated in Fig. 2. For the determination of the LOD, *y*_LOD_ = initial value (A1) + 3 × standard deviation of the blank (SD_blank_) was chosen as it yields a more realistic LOD in contrast to the often-used linear approach. Already the small liposomes showed here significant improvement of the LOD with 4 pg mL^−1^ IL-6 in comparison to colloidal gold with an LOD of 0.025 ng mL^−1^ when detected photometrically (Fig. S5) with similar resolution (slope_190 nm_ = 0.7 mL pg^−1^, slope_colloidal gold_ = 0.5 mL pg^−1^). Switching to large liposomes showed sensitivity enhancement not only by an even lower LOD (1 pg mL^−1^) but also a significantly increased slope of 3.4 mL pg^−1^ (Fig. S5) was obtained, which is due to the increased inner volume of the liposomes and thus elevated amount of dye present (slopes were determined from the linear region). Furthermore, the large liposome approach had a dynamic range of almost three orders of magnitude. Even when measured photometrically in serum, the liposomes outperform commercial colloidal gold with slightly increased LOD towards the buffer conditions (LOD_190 nm_ = 23 pg mL^−1^, LOD_350 nm_ = 7 pg mL^−1^, LOD_colloidal gold_ = 81 pg mL^−1^) (Fig. 3 a).

Since the strategy to achieve a lower LOD using inherently fluorescent liposomes was not successful, a system was developed in which liposomes were bound to test, and control lines were lysed through an additional step in order to harness their superior fluorescent capability. In addition, pretests in a microtiter plate approach showed that the well-known fluorescence enhancement of SRB in human serum [18] could assist in enhancing the sensitivity (Online Resource 1 – ESM 2.4, Fig. S3, Table 3). Yet, with the current assay design, it is not possible to take advantage of this in the LFA format (Fig. S4). We assume that this is due to the absence of a bulk aqueous enviroment on the test strip which does not allow for a polarity change when introducing HSA into the system. Furthermore, surprisingly, the fluorescence approach did not yield a lower LOD (0.2 ng mL^−1^) in contrast to the photometric approach. This is most likely due to the high background signal and the inherently introduced error of the additional procedural steps. The background signal can be lowered by changing the assay procedure and preapplying the liposomes to the conjugate pads. However, as can be seen in Fig. 3, the lack in preincubation time compensates any benefit obtained through lower background signals. Specifically, it led to an increased LOD not only with the liposomes but also with the AuNP (0.1 ng mL^−1^) (Fig. 3 b). In the future, other membrane materials that allow for more interactions such as slow running membranes (CN150) (Fig. S6) will be investigated along with developing an effective dehydration strategy for the large liposomes. When referencing to already published articles, our results are impressive as we show here several versions of LFA procedures that can be operated with undiluted real-world samples and yield already in its easiest form (photometrically) exceptional sensitivity with only 36 µL sample volume, whereas in literature often samples with only 10% serum as matrix were employed, and typically more sample volume is needed (Table 3). Overall, only a few articles are published which developed LFA-based IL-6 detection (Table 3), and most of them are limited to academic studies only and require sophisticated detection devices. Here, on the contrary, a commercially ready system was used and enhanced by refined liposomal reporter probes which, similarly to AuNP, can be used for a multitude of immune-LFAs. Already the photometric readout competes with the sensitivity of most of the reported LFAs and the commercial colloidal gold approach. We envision simultaneous photometrical or fluorescent application in the future providing LODs in the LFA format that can rival signal generation in microtiter plate assays.

**Table 3:** Recently published techniques for sensitive detection of interleukin 6 with immuno-LFAs

Detection method	LOD	Matrix	Special remarks	Ref
**Fluorescence** (Eu-NP^a^)	0.37 pg mL^−1^	Buffer, human serum	70 µL sample LFA run 15 min, commercial strip reader	[19]
**Fluorescence** Quantum dots (QD)	100 pM (2.1 ng mL^−1^)^b^	Buffer, 10% serum	LFA run 20 min 100 µL sample, multiplex, protype detector with UV-LED	[20]
**Photon-upconverion** (UCP)^c^	n. a	Diluted whole blood	50-fold diluted benchtop reader, UPCON, Labrox	[21]
**SERS**^d^ Au/Au core satellite nanoparticels^e^	n. a	PBS	Proof of principle, multiplex, non-commercial portable SERS reader	[22]
**Fluorescence** (fluorescent microspheres^f^)	7.15 pg mL^−1^ 48.5 pg mL^−1^	Human plasma, hydrogel samples	Up to 33 µL, extra washing steps, commercial strip reader	[23]
**Fluorescence** (Near-infrared dye^g^)	4 pg mL^−1^ (182 fmol L^−1^)	10% human plasma	75 µL sample, LFA run ≥ 15 min, benchtop image scanner	[24]
**Fluorescence** Quantum dots (QD)	4.5 pM (0.09 ng mL^−1^)	Buffer, 10% human serum	120 µL sample, LFA run 20 min benchtop image scanner	[25]
**Photometry** (commercial colloidal gold)	0.025 ng mL^−1^ (buffer) 0.081 ng mL^−1^ (HS)	Buffer, 100% human serum (HS)	36 µL sample LFA run 15 min, commercial strip reader	This work
**Photometry** (dye-loaded liposomes)	1 pg mL^−1^ (buffer) 7 pg mL^−1^ (HS)	Buffer, 100% human serum (HS)	36 µL sample LFA run 15 min, commercial strip reader	This work

^a^Europium(III) chelate–doped nanoparticles; ^b^molecular weight of 21 kDa for IL-6 was presumed; ^c^up-converting phosphor nanoparticles; ^d^surface enhanced Raman scattering: ^e^core functionalized with Raman-active 4-nitrothiophenol for IL-6 or thio-2-naphthol for IL-8; ^f^FluoSpheres®; fluorophore-doped particles (200 nm); ^g^IRDye 800CW (Li-Cor Biosciences)

**Fig. 4:**
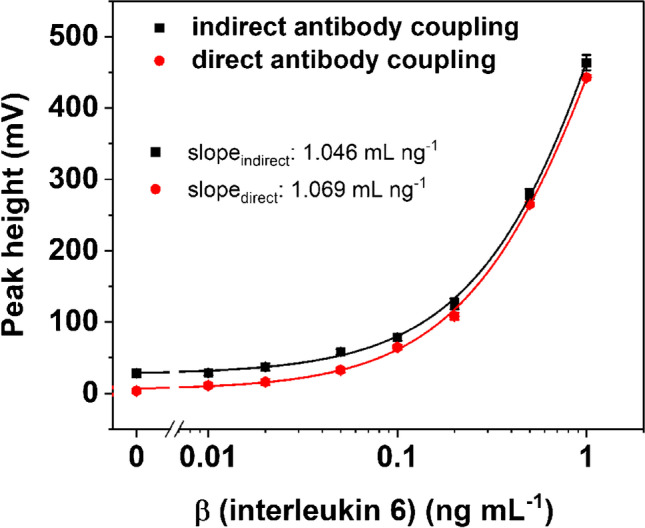
Performance test of universal streptavidin-modified liposomes towards direct-coupled small liposomes with anti-interleukin 6 IgG (< IL-6 >), four-parameter logistic fit with *R*^2^ = 0.9970 (black) and *R*^2^ = 0.9993 (red). Fifty microliters of a mixture of IL-6 and liposomes (40 mOD per test) in running solution were applied to the test strip (< IL-6 > test line), test run for 15 min. Streptavidin-liposomes were mixed with anti-IL-6-biotin (equaling 0.2 µg anti-IL-6-biotin per test) and IL-6; photometric measurement was done at *λ*_max_ = 520 nm; data are presented as mean ± SD (error bar) with *n* = 3, y_LOD_ = A1 + 3 × SD_blank_; slope derived from four-parameter logistic fit function

In order to render a truly universal liposome label approach, we compared directly coupled < IL-6 > liposomes to those indirectly coupled to streptavidin-coated liposomes via < IL-6 > -biotin. Such a universal reporter probe bypasses stability issues typically encountered with antibodies extending the overall shelf life of this reagent. We obtained no significant difference between the two strategies, as similar signal intensities, equal slopes (slope_indirect_: 1.046 mL ng^−1^, slope_direct_: 1.069 mL ng^−1^) and sensitivities (LOD_direct_: 0.019 ng mL^−1^, LOD_indirect_: 0.026 ng mL^−1^) were obtained with the direct and indirect approaches (Fig. 4).

### Stability study and liposome dehydration

**Fig. 5:**
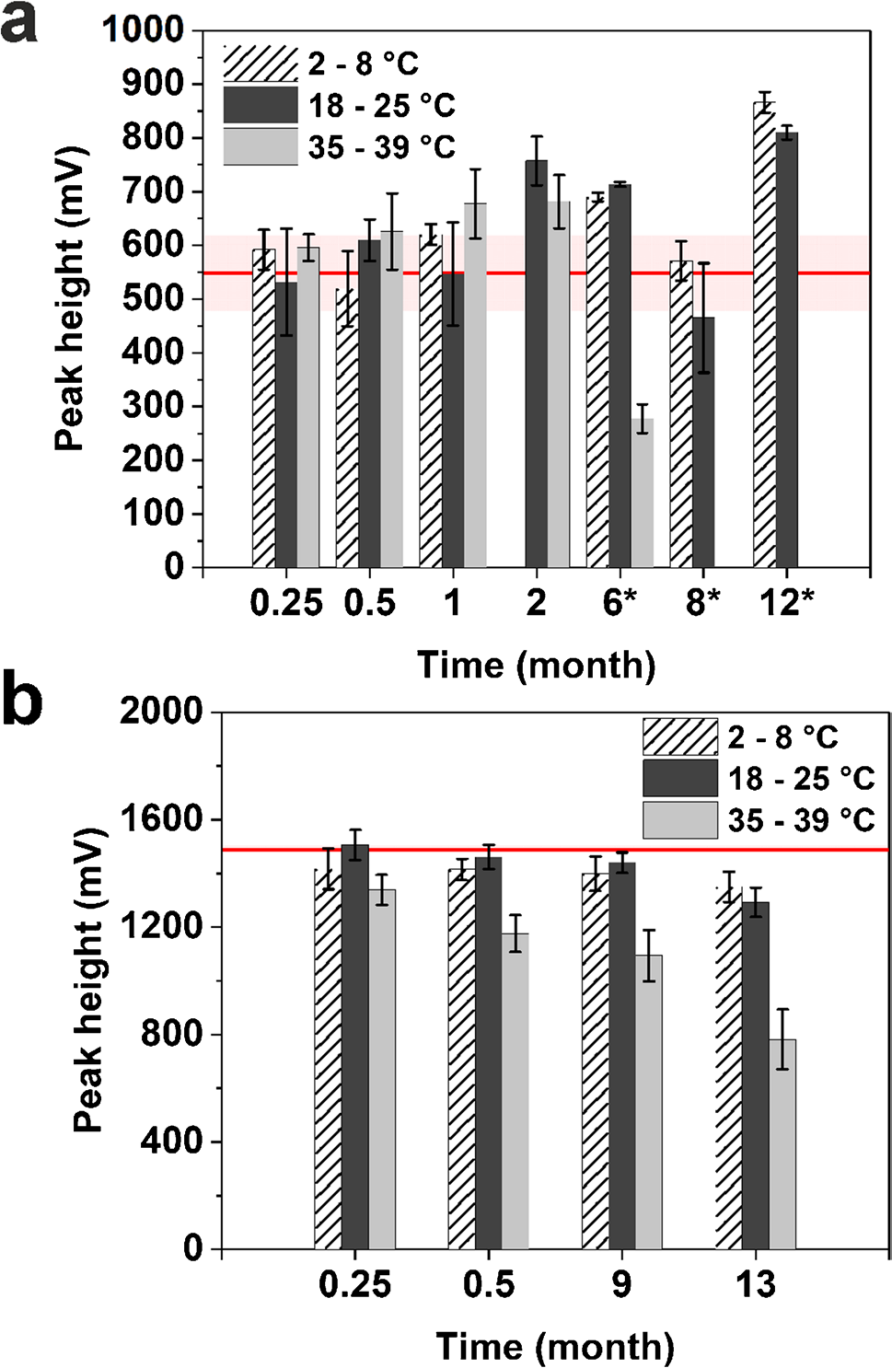
Long-term stability of small streptavidin-liposomes in solution **a** or dehydrated on a test strip **b** before test run on LFA strips with biotin test line; red line indicates initial response at time point zero. Liposomes were diluted to 25 mOD per test in 90 µL **a**, test run for 5 min; in **b**, liposomes with 25 mOD per test were dehydrated on test strip and rehydrated by 50 µL running buffer, test run for 15 min. Photometric measurement was done at *λ*_max_ = 520 nm; data are presented as mean ± SD (error bar) with *n* = 5; times marked with an asterisk equals triplicates, reference line indicates initial response

Since the direct and indirect coupling approach performed similarly in the assay, the stability study was conducted with streptavidin-modified liposomes. In the end, these would be favored over the direct coupling approach in a commercial application as they can function as a generic label. These universal streptavidin-modified liposomes that are independent from the durability of the antibody itself were used in a long-term stability study assessing their stability in solution as well as in dehydrated form on the conjugate pad. Testing was conducted in a simple streptavidin–biotin assay with the small (190 nm) streptavidin-modified liposomes. These liposomes remained stable in solution for at least 12 months at 2–8 °C and even 2 months at elevated temperatures up to 39 °C (Fig. 5 a). The results from such an accelerated stability study translate roughly into 8 years when stored at 4 °C [26].

**Fig. 6:**
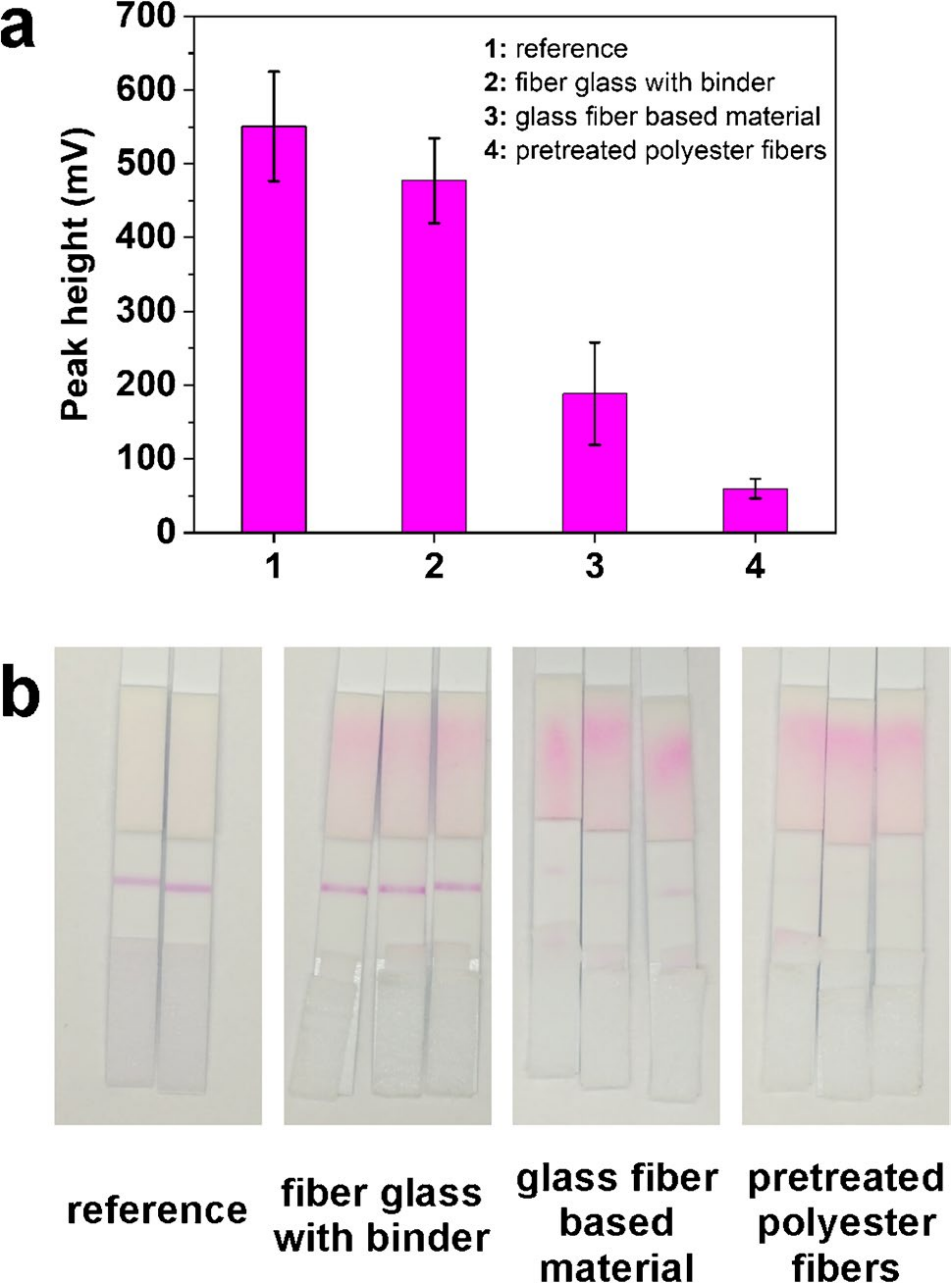
Evaluation of different conjugate pad materials for small liposome conjugates **a** obtained signal intensities and **b** real images. Test strips were prepared with 5 µL liposome dilution in conjugate pad buffer (80 mOD per test); test run for 5 min in 100 µL running buffer; photometric measurement was done at *λ*_max_ = 520 nm; data are presented as mean ± SD (error bar) with n ≥ 2

Dehydration of the liposomes onto the conjugate pad was mainly motivated by the desire to reduce the number of assay steps and reagents needed to perform a LFA. In a first attempt, we identified the most suitable conjugate pad materials by simply drying our liposomes on different pad materials in HSS buffer at room temperature and 50% air humidity for 1 h. The liposomes retained approximately 80% of the liquid signal when dried on the conjugate pad made of fiber glass with binder (Fig. 6), whereas all other pad materials studied showed meager performance (recovery < 50%). As manufacturers frequently pretreat membranes and pad material with inter alia detergents and typically use confidential binder formulations, this observation is not surprising and rather emphasizes the need for prescreening of LFA materials within the development process. For the follow-up long-term stability study, liposomes were dispensed on the fiber glass conjugate pad with binder and dried in a drying cabinet at 37 °C for 1 h. After assembling, the test strips were stored at 2–8 °C, 18–25 °C, and at 35–39 °C and tested periodically to assess the reduction in the response. The test strips remain stable for at least 9 months at temperatures between 2 and 25 °C with no significant reduction in the initial signal response and only a minor signal drop (< 10%) when retested after 13 months (Fig. 5 b).

Harrigan and colleagues [27] stated that the stability of vesicles critically depends on the vesicle size with the result that smaller systems are most stable. We made similar observations, as currently only the small liposomes straightforwardly tolerate the dehydration process and were hence used for the long-term stability study. Dehydration of the large liposomes is currently under investigation as present data indicates that the liposomes are not fully destroyed (data not shown). Optimization of the drying conditions most likely provides the desired remedy, where we will apply protecting sugars as suggested previously by Martorell and colleagues [28]. They observed decreased recoveries for the larger liposomes as well but to a lesser extent (approx. 60% in contrast to 75% recovery with small liposomes). The results are not directly comparable as support material and size determination vary but it points to prosper when refining the dehydration conditions for our large liposomes.

## Conclusion

The established commercial LFA system with colloidal gold for the detection of IL-6 shows already good sensitivity with 0.025 ng mL^−1^. However, by replacing the gold nanoparticles with refined dye–loaded liposomes, we were able to improve the sensitivity by over one order of magnitude to just 1 pg mL^−1^ with simple photometric detection. Furthermore, utmost care was taken to ensure that the liposomes are easily mass-producible, could be dehydrated on the LFA membrane itself, and hence could be applied in the same, straightforward, simple, and easy-to-use LFA strategy that is so desirable. Further improvement of the LOD through fluorescent detection approaches, however, is not as easy to accomplish. Inherently, fluorescent liposomes do not provide enough signal intensity and those that require an additional process step, as the dye has to be released from the liposome prior to detection, unfortunately compensate any gained signal intensity at the LOD by higher background signals and less reproducibility due to the additional assay steps. Thus, while in a refined environment such as a microtiter plate, improved LODs can be obtained through fluorescence detection of these liposomes; this is not as easily translated to a robust, commercially ready LFA approach. More experiments are needed to lower background signals, improve analyte-liposome interactions, and hence lower the LOD effectively. However, already now the increased signal intensity afforded by the fluorescent liposomes will assist in the development of less sophisticated detection devices. Expanding on the applicability of these new reporter probes, our universal liposomes, which maintain sensitivity levels of directly conjugated liposomes, show remarkable long-term stability when stored in solution and dehydrated on a test strip of at least 1 year. These adaptable liposomes can easily be transferred to any other analyte of interest manifesting them as a true alternative to standard colloidal gold. With this, a highly flexible and supersensitive toolset is provided for tailored assay development. Furthermore, in light of the importance of IL-6 detection with infectious diseases such as COVID-19, the here presented liposome-based LFA indicates that liposomes will rival the prevalence of colloidal gold as a benchmark in LFA analysis.

## Supplementary Information

Below is the link to the electronic supplementary material.

Supplementary file1 (DOCX 1162 KB)

## Data Availability

Electronic supporting material is available online.
