# Supplementary material for: Highly sensitive interleukin 6 detection by employing commercially ready liposomes in an LFA format
Source: Anal Bioanal Chem. 2021 Nov 13;414(10):3231–41. doi: 10.1007/s00216-021-03750-5 (PMC8590136; doi:10.1007/s00216-021-03750-5)
Supplement: Supplementary file 1 — Supplementary file1 (DOCX 1162 KB) [file 216_2021_3750_MOESM1_ESM.docx]

**Electronic Supporting Material**

**Journal: Analytical and Bioanalytical Chemistry**

**Highly sensitive interleukin 6 detection by employing commercially ready liposomes in an LFA format**

Simone Rink^a^, Barbara Kaiser^b^, Mark-Steven Steiner^b^, Axel Duerkop^a^, Antje J. Baeumner^a,^*

**^a^Institute of Analytical Chemistry, Chemo- and Biosensors, University of Regensburg, Universitätsstraße 31, 93053 Regensburg, Germany**

^b^Microcoat Biotechnologie GmbH, 82347 Bernried am Starnberger See, Germany

***Author for correspondence:**

**Email: antje.baeumner@ur.de**

**Phone: +49 941 943 4065**

**Postal address:**

**Antje Baeumner**

**Universität Regensburg**

**93040 Regensburg**

**Germany**

1. **Outline**

[1. Experimental Section 2](#_Toc84537273)

[1.1. Chemicals and consumables 2](#_Toc84537274)

[1.2. Synthesis of sulforhodamine B liposomes. 2](#_Toc84537275)

[1.3. Matrix effect evaluation. 3](#_Toc84537276)

[1.4. Lateral flow assay procedure. 3](#_Toc84537277)

[2. Results 3](#_Toc84537278)

[2.1. Size adjustment of sulforhodamine B liposomes through variation of extrusion parameters 3](#_Toc84537279)

[2.2. Additional information to large sulforhodamine B liposomes with varying encapsulation concentration 4](#_Toc84537280)

[2.3. Pretests for antibody coupling to liposomes 4](#_Toc84537281)

[2.4. Matrix effect on fluorescence signal (MTP-based) 6](#_Toc84537282)

[2.5. Photometric and Fluorescence IL-6 Assay in running buffer 7](#_Toc84537283)

[3. References 9](#_Toc84537284)

# **Experimental Section**

# **Chemicals and consumables**

Standard chemicals were purchased from Sigma Aldrich/Merck and used without purification. Chloroform and methanol were purchased from VWR chemicals (Germany). Milk powder, cholesterol, Sephadex® G 50, sucrose, sodium azide, glycine (purity >99.7 %), sodium hydroxide, TWEEN®20, Whatman Nucleopore™ Track-Etched membranes 1.0 µm, 0.4 µm and 0.2 µm, 19 mm diameter were purchased from Sigma Aldrich/Merck (Germany). Potassium hydrogen carbonate, *n*-octyl-*β*-*D*-glucopyranoside (CN23.2), dialysis tube Spectra/Por© 4 (2718.1) MWCO (12-14 kDa) were obtained from Carl Roth (Karlsruhe, Germany). Sepharose CL-4B was purchased from Cytiva Europe GmbH (Freiburg, Germany). Phosphorous standard was purchased from Bernd Kraft GmbH (Duisburg,Germany) and HNO_3_ from Fisher Scientific GmbH (Schwerte, Germany). Synthetic sweat was purchased from synthetic urine (Eberdingen-Nussdorf, Germany). For all experiments ultrapure water was used.

# **Synthesis of sulforhodamine B liposomes**

The synthesis was done according to the procedure in the main article by using DPPC (29.58 mg, 40.3 µmol), DPPG (15.64 mg, 21.0 µmol), cholesterol (19.99 mg, 51.7 µmol) and *N*-glutaryl-DPPE (6.2 mg, 7.0 µmol). After rotary evaporation the remaining solution was split into 7 fraction and extruded at 65 °C. Each fraction was extruded with varying parameters with regard to the applied membranes (no membrane, only 1 µm, 1 µm and 0.4 µm or 1 µm, 0.4 µm and 0.2 µm) and the amount of extrusion steps (11 or 21 steps) using a mini extruder (Avanti Polar Lipids, Inc.). Each fraction was purified first by size‑exclusion chromatography with Sephadex® G‑50 as stationary phase (column size: 1 cm x 6 cm) and HSS buffer (10 mmol L^‑1^ HEPES, 200 mmol L^‑1^ sodium chloride, 200 mmol L^‑1^ sucrose, 0.01 wt% sodium azide), pH 7.5, osmolality 0.643 osmol kg^-1^ as mobile phase. Additionally, the liposomes were dialyzed against HSS buffer until the dialysis buffer remains colorless before determining the respective hydrodynamic diameter via DLS, phospholipid concentration via ICP-OES and zeta-potential.

# **Matrix effect evaluation**

A series of liposome dilutions were prepared in HSS buffer containing 30 mmol L^-1^ *n*-octyl-*β*-*D*-glycopyranosid and 10 % (v/v) of the respective matrix. 100 µL of each dilution was measured in a black medium-binding microtiter plate (Greiner BioOne, Frickenhausen, Germany) with a Synergy Neo 2 microplate reader from BioTek (Bad Friedrichshall, Germany). Fluorescence measurement was performed with λ_ex_ = 530 nm (10 nm), λ_em_ = 590 nm (10 nm) and gain 100 and a reading height of 4.5 mm.

# **Lateral flow assay procedure**

The LFAs were similarly performed to the procedure described in the main manuscript.

# **Results**

# **Size adjustment of sulforhodamine B liposomes through variation of extrusion parameters**

Here, the size of the liposomes was adjusted by varying the applied extrusion membranes and the extrusion steps. Whereas increasing extrusion steps generally lead to a lower polydispersity index (PdI), only extrusion to solely a 1 µm membranes leads to a significant size difference compared to those extruded through 0.4 µm and 0.2 µm membranes (Table S 1). This is also depicted in **Fig. S 1** with more confined peaks for the fractions extruded with 21 steps and a shift of the peak for the fractions extruded through 0.4 µm and 0.2 µm membranes to smaller size distributions.

**
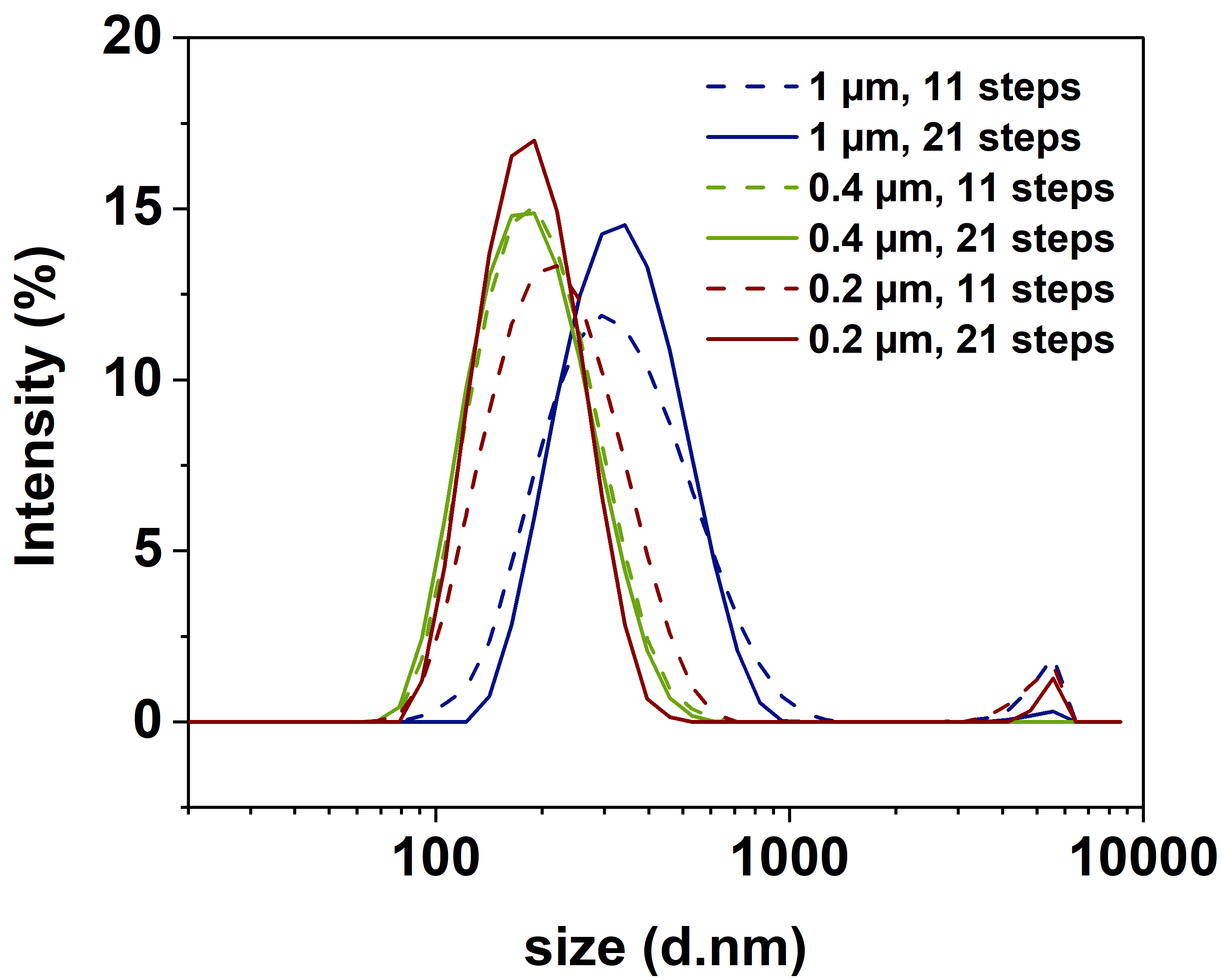
**

**Fig. S 1** Hydrodynamic diameter of 150 mmol L^-1^ sulforhodamine B liposomes synthesized with varying extrusion membranes and extrusion steps. DLS was carried out with a 1:100 dilution of liposomes in HSS buffer, data are presented as mean with n = 3, error bar not shown

**Table S 1** Size parameters obtained for 150 mmol L^-1^ sulforhodamine B liposomes synthesized with varying extrusion conditions

| **Extrusion parameters** | **Size by Intensity**  **(nm)** | **Size by Number**  **(nm)** | **Z-average**  **(nm)** | **Polydispersity index^a^** |
| --- | --- | --- | --- | --- |
| **w/o extrusion^b^** | n. a. | n. a | n. a. | n. a. |
| **1 µm,**  **11 steps** | 356 ± 161 | 231 ± 111 | 323 | 0.20 |
| **1 µm,**  **21 steps** | 355 ± 132 | 277 ± 110 | 309 | 0.15 |
| **0.4 µm,**  **11 steps** | 205 ± 76 | 136 ± 52 | 189 | 0.14 |
| **0.4 µm,**  **21 steps** | 198 ± 74 | 131 ± 49 | 175 | 0.14 |
| **0.2 µm,**  **11 steps** | 232 ± 92 | 146 ± 63 | 219 | 0.16 |
| **0.2 µm,**  **21 steps** | 193 ± 60 | 141 ± 48 | 196 | 0.10 |

DLS was carried out with a 1:100 dilution of liposomes in HSS buffer; ^a^polydispersity (PdI) was calculated for the corresponding peak according to PdI = (σ_size_ /size)^2^, here the size by intensity values were used [S1], ^b^no reliable dataset was obtainable for liposomes without extrusion as they show too high variation in size; data are presented as mean ± SD with n = 3

# **Additional information to large sulforhodamine B liposomes with varying encapsulation concentration**

**Table S 2** lists the characteristics of the synthesized large sulforhodamine B liposomes with varying SRB encapsulation concentrations.

**Table S 2** Characteristics of large sulforhodamine B liposomes

| **encapsulant**  **concentration** | **hydrodynamic**  **diameter^a^**  **(nm)** | **ζ‑potential (mV)** | **Polydispersity index** | **I_lysed_^b^ × 10^3^**  **(a. u.)** | **I_intact_^c^**  **(%)** |
| --- | --- | --- | --- | --- | --- |
| 10 mmol L^-1^ | 457 ± 170 | -40 ± 3 | 0.24 ± 0.01 | 12.5 ± 0.5 | 0.66 ± 0.03 |
| 50 mmol L^-1^ | 290 ± 146 | -40 ± 1 | 0.23 ± 0.01 | 49.3 ± 0.9 | 0.259 ± 0.005 |
| 150 mmol L^-1^ | 355 ± 132 | -29.7 ± 0.7 | 0.18 ± 0.02 | 66 ± 1 | 0.152 ± 0.002 |

^a^size by intensity of a 1:100 dilution, ^b^I_intact_ was obtained by diluting liposomes to 100 µmol L^-1^ total lipid in HSS buffer (100 µL) and I_lysed_ by diluting the liposomes in 30 mmol L^-1^ *n*-octyl-*β-D*-glycopyranoside in HSS buffer, ^c^I_intact_ = I_intact_/I_lysed_ **×** 100, data are presented as mean ± SD with n = 3

# **Pretests for antibody coupling to liposomes**

To define ideal coupling conditions antibody coupling was pretested with the model antibody anti-digoxigenin IgG (<Dig>). The liposomes contain 6 mol% of lipids bearing a glutaryl group available for EDC/NHS coupling chemistry, which limits the overall surface coverage obtainable in comparison to AuNPs. Nonetheless, with a coupling ratio of 1:17:42:0.17 (n(COOH):n(EDC):n(NHS):n(antibody)), the small liposomes outperformed commercial AuNPs (**Fig. S 2 a, b**). In addition, a visibly lower limit of detection (**Fig. S 2** **c**) at 1 ng mL^-1^ was obtained for liposomes, and at 10 ng mL^-1^ for AuNPs. The overall lower signal intensity and earlier saturation obtained for the liposomes was caused by the maximally available loading of the surface, i.e. limited by the 6 mol% glutaryl groups. Fine-tuning of the final loading of the liposomes with <Dig> can overcome the lower signal intensity and saturation issue. However, this was not our primary focus in the present study.


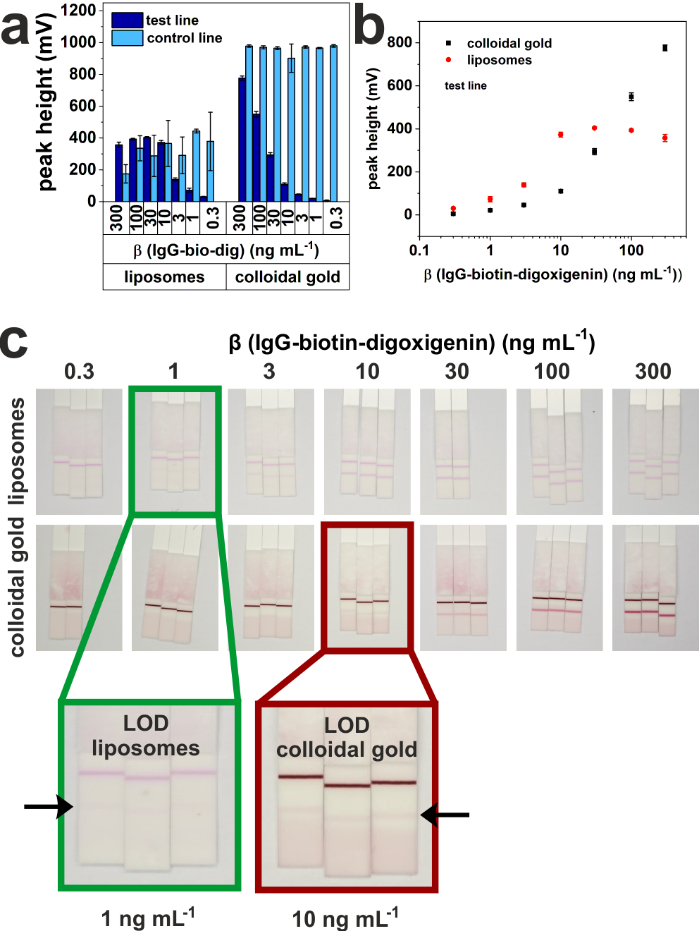


**Fig. S 2** Photometric titration of IgG-biotin-digoxigenin with anti-digoxigenin labeled liposomes in comparison to commercial gold nanoparticle with (a) illustrating the test and control line signals, (b) test line dose-response curve with small liposomes in direct comparison to colloidal gold and (c) images of lateral flow assays with visible limit of detection. Particles were preincubated (5 min) in running solution (85 µL IgG-biotin-digoxigenin dilution, 10 µL particles (80 mOD per test)), test run for 5 min, photometric measurement was done at λ_max_ = 520 nm, data are presented as mean ± SD (error bar) with n ≥ 2

# **Matrix effect on fluorescence signal (MTP-based)**

For IL-6, human serum is the state-of-the-art matrix. It is proposed in literature that interaction of fluorophores with human serum albumin (HSA) can result in enhanced fluorescence due to non-covalent interaction of the fluorophore with binding side I in HSA [S2]. Kitamura and colleagues [S3] intensively studied the interaction between HSA and SRB and proposed that it binds through hydrophobic interaction to the Sudlow site I of HSA and the accompanied change to a less polar environment benefits the fluorescence intensity of sulforhodamine B. Exploiting this fluorescence enhancement was intended to increase sensitivity not only in the microtiter plate approach but also in the LFA when measuring in human serum. However, as we also accomplished a universal liposome label, various analytes become accessible. Thus, we studied the influence of different matrices toward their effect on the fluorescence signal. We obtained fluorescence enhancement in human serum and in the presence of HSA, whereas no fluorescence enhancement was obtained in milk, synthetic sweat, fetal bovine serum and in the presence of BSA (**Fig. S 3**) which is also mirrored in the obtained LODs.


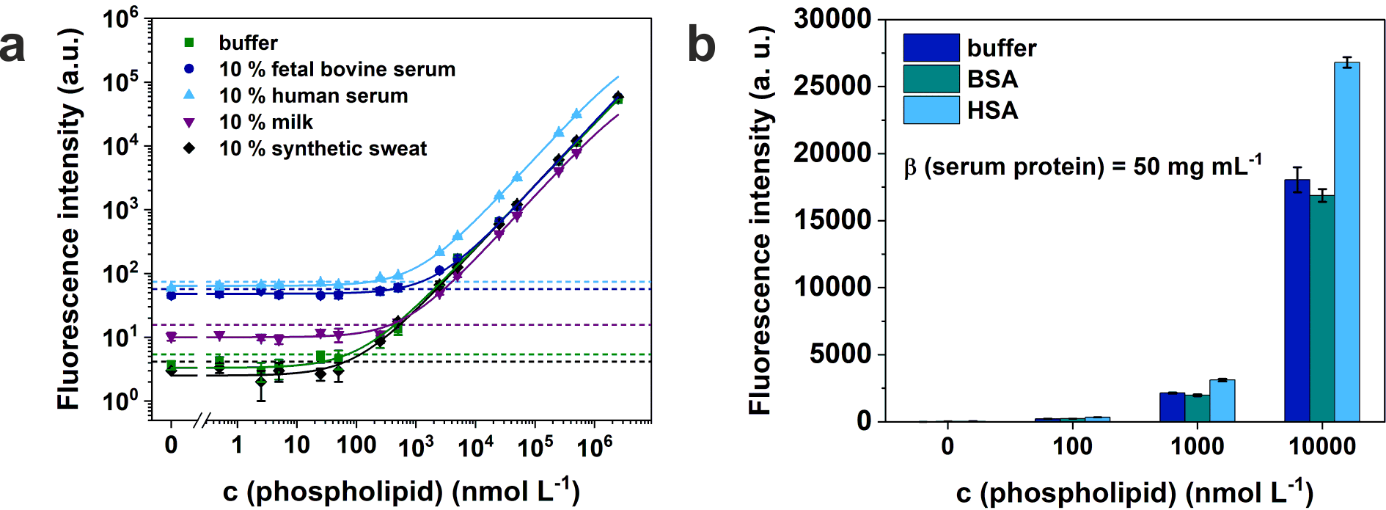


**Fig. S 3** Dose-response curve of fluorescence liposomes in various matrices and HSS buffer in (a) and enhancement obtained in presences of bovine serum albumin (BSA) and human serum albumin (HSA) in (b) and in HSS buffer. Fluorescence measurement was performed with λ_ex_ = 530 nm (10 nm), λ_em_ = 590 nm (10 nm) and gain 100 (RH 4.5), as fitting function, the four-parameter logistic fit from OriginLab 2020 was applied, data are presented as mean ± SD (error bar) with n = 3

As the binding site I in bovine serum albumin (BSA) is altered with an additional leucin residue, current assumptions are that this binding site is blocked in BSA and no enhancing proteins are present in the other tested matrices and thus fluorescence enhancement is only observed with HSA [S2]. The obtained LODs with liposomes in the different matrices are listed in **Table S 3**.

**Table S 3** Matrix effect on fluorescence signal

| Matrix^a^ | LOD  (pmol L^-1^) |
| --- | --- |
| HSS buffer | 59 |
| fetal bovine serum | 515 |
| human serum | 105 |
| milk | 300 |
| synthetic sweat | 70* |

LOD: limit of detection ( y_LOD_ = A1 + 3 × SD_blank_), *LOD was calculated from 0.5 nmol L^-1^ phospholipid concentration sample

In solution a sensitivity enhancement with regard to other complex matrices (FBS or milk) was obtained. Unfortunately ,the enhancement effect from HSA (**Fig. S 3**) in the microtiter plate was not obtained in the LFA approach. **Fig. S 5** shows IL-6 titration with 350 nm liposomes in running buffer and serum to determine enhancement of fluorescence in human serum on an LFA and encountered sensitivity gain.

**
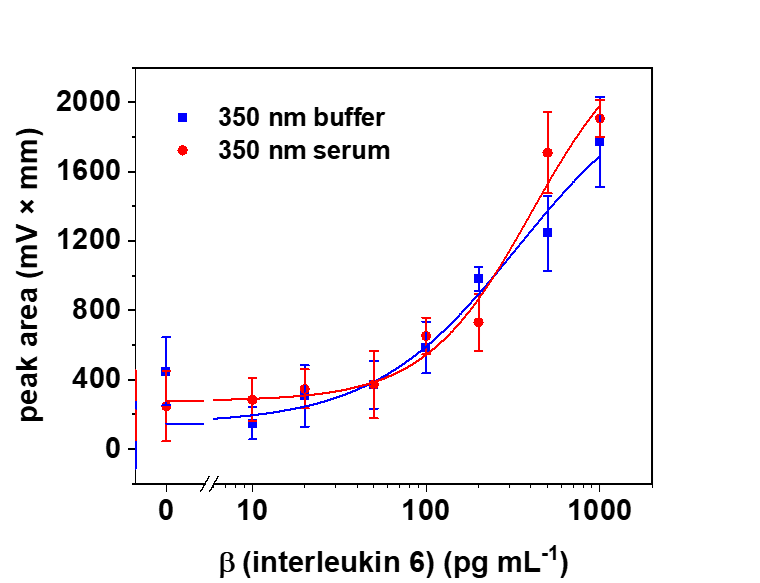
**

**Fig. S 4** Titration of IL-6 with large liposome conjugates after lysis in running buffer and serum, fluorescence signal was recorded with λ_ex_ = 470 nm, λ_em_ = 600 nm, data are presented as mean ± SD (error bar) with n = 3, four-parameter logistic fitting with Origin2020

# **Photometric and Fluorescence IL-6 Assay in running buffer**


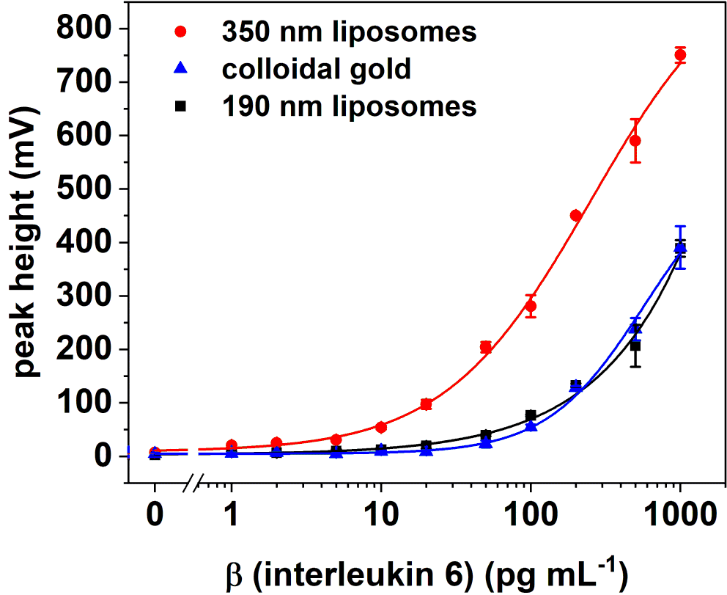


**Fig. S 5** Titration of IL-6 with large and small liposome conjugates benchmarked to colloidal gold. (a) Photometric detection in running buffer with preincubation of liposomes (5 min) with IL-6 and anti-IL6-biotin IgG in running solution, photometric measurement was done at λ_max_ = 520 nm, data are presented as mean ± SD (error bar) with n = 3, four-parameter logistic fitting with Origin2020, y_LOD_ = A1 + 3 SD_blank_

**Fig. S 6** illustrates the pretest results for fluorescence and photometric IL-6 titrations with LFAs where the detection particles are directly dropped on the conjugate pad and the LFA run is immediately starting. Here, a slow running membrane (CN150) and a fast-running membrane (CN95) were tested.


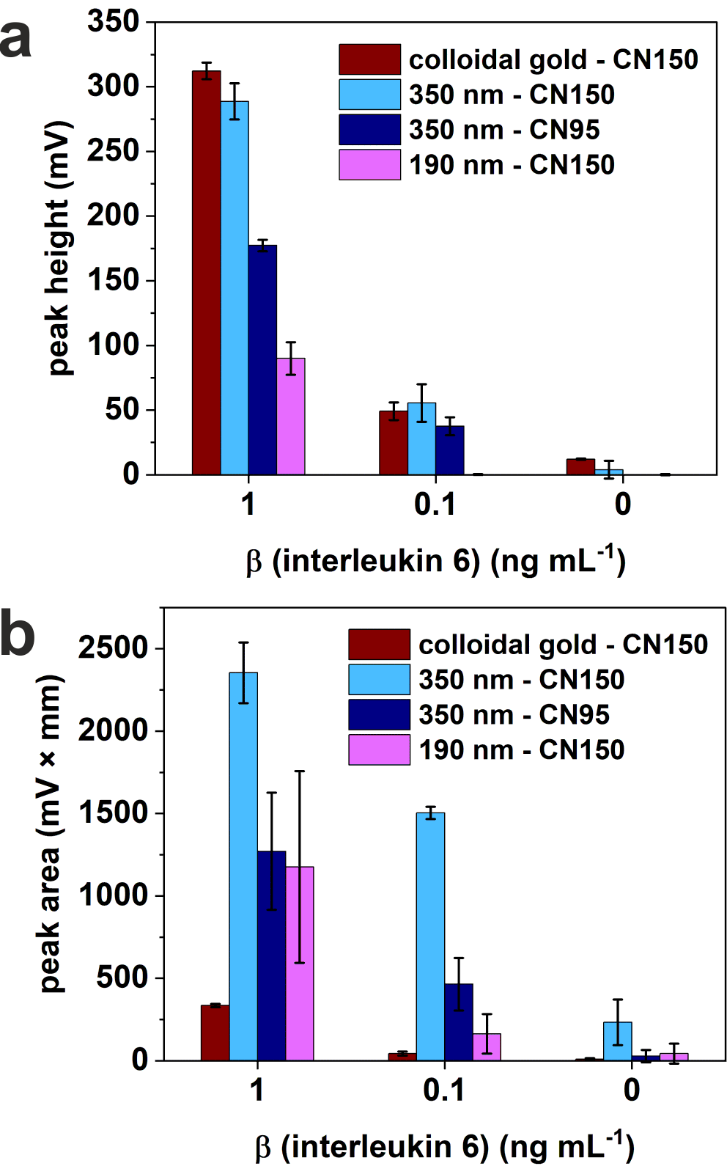


**Fig. S 6** Procedural pretests for IL-6 titration with large and small liposome conjugates benchmarked to colloidal gold. (a) Photometric detection and (b) fluorescence detection of liposomes after lysis benchmarked to colloidal gold. Liposomes were applied to conjugate pad and the assay performed in running buffer, photometric measurement was done at λ_max_ = 520 nm, fluorescence signal was recorded with λ_ex_ = 470 nm, λ_em_ = 600 nm, data are presented as mean ± SD (error bar) with n = 3

# **References**

S1. Raval N, Maheshwari R, Kalyane D, Youngren-Ortiz SR, Chougule MB, Tekade RK. Importance of Physicochemical Characterization of Nanoparticles in Pharmaceutical Product Development. In: Tekade RK, editor. Basic fundamentals of drug delivery. London, United Kingdom, San Diego, CA: Academic Press an imprint of Elsevier; 2019. pp. 369–400.

S2. Fan J, Sun W, Wang Z, Peng X, Li Y, Cao J. A fluorescent probe for site I binding and sensitive discrimination of HSA from BSA. Chem. Commun. (Cambridge, U.K.). 2014; https://doi.org/10.1039/c4cc03778b

S3. Kitamura M, Murakami K, Yamada K, Kawai K, Kunishima M. Binding of sulforhodamine B to human serum albumin: A spectroscopic study. Dyes Pigm. 2013; https://doi.org/10.1016/j.dyepig.2013.06.011
